# Point-of-care, smartphone-based, dual-modality, dual-view, oral cancer screening device with neural network classification for low-resource communities

**DOI:** 10.1371/journal.pone.0207493

**Published:** 2018-12-05

**Authors:** Ross D. Uthoff, Bofan Song, Sumsum Sunny, Sanjana Patrick, Amritha Suresh, Trupti Kolur, G. Keerthi, Oliver Spires, Afarin Anbarani, Petra Wilder-Smith, Moni Abraham Kuriakose, Praveen Birur, Rongguang Liang

**Affiliations:** 1 College of Optical Sciences, The University of Arizona, Tucson, Arizona, United States of America; 2 Mazumdar Shaw Medical Centre, Bangalore, India; 3 Mazumdar Shaw Medical Foundation, Bangalore, India; 4 Biocon Foundation, Bangalore, India; 5 KLE Society’s Institute of Dental Sciences, Bangalore, India; 6 Beckman Laser Institute, University of California, Irvine, Irvine, California, United States of America; Texas A&M University, UNITED STATES

## Abstract

Oral cancer is a growing health issue in a number of low- and middle-income countries (LMIC), particularly in South and Southeast Asia. The described dual-modality, dual-view, point-of-care oral cancer screening device, developed for high-risk populations in remote regions with limited infrastructure, implements autofluorescence imaging (AFI) and white light imaging (WLI) on a smartphone platform, enabling early detection of pre-cancerous and cancerous lesions in the oral cavity with the potential to reduce morbidity, mortality, and overall healthcare costs. Using a custom Android application, this device synchronizes external light-emitting diode (LED) illumination and image capture for AFI and WLI. Data is uploaded to a cloud server for diagnosis by a remote specialist through a web app, with the ability to transmit triage instructions back to the device and patient. Finally, with the on-site specialist’s diagnosis as the gold-standard, the remote specialist and a convolutional neural network (CNN) were able to classify 170 image pairs into ‘suspicious’ and ‘not suspicious’ with sensitivities, specificities, positive predictive values, and negative predictive values ranging from 81.25% to 94.94%.

## 1 Introduction

Oral cancer incidence and death rates are rising in low- and middle-income countries (LMIC) [1–5]. As of 2012, 65% of new oral cancer cases and 77% of oral cancer deaths occurred in LMIC [6] with a five year survival rate under 50% in some countries [7].

Oral cancer development is increased by a number of lifestyle choices including tobacco [8, 9] and alcohol use [10]. Particularly in Asia, betel quid (or paan) chewing (with or without tobacco [11, 12]) increases rates of oral squamous cell carcinoma (OSCC) and oral submucous fibrosis (OSMF) [13–21]. Betel quid (typically consisting of betel leaf, areca nut, slaked lime, and possibly tobacco [22]) was identified as a contributer to increased oral cancer incidence as early as 1902 [23]. Despite the risk of developing oral cancer, psychostimulating qualities keep betel quid popular [22, 24–26].

High-risk populations living in remote areas with limited access to healthcare infrastructure are in need of low-cost, easy-to-use medical imaging devices to enable early diagnosis with increased sensitivity as early diagnosis is well correlated with higher survival rates [7]. Conventional visual examinations achieve sensitivities around 60% with specificity over 98.5% [27] but require visible lesions, possibly delaying diagnosis.

Autofluorescence imaging (AFI) is an alternate detection technique using changes in the radiant exitance of oral tissue fluorescence when illuminated at 400–410 [28–30] to discriminate potential oral malignant lesions, removing the requirement of the lesion being visible [19, 28, 31–41]. Increasing dysplasia results in a decreased fluorescence signal from changes in endogenous fluorophores and increased absorption from hemoglobin [29, 33, 34, 42–44]. Carcinogenesis affects cellular structure, breaking down the collagen and elastin cross-linking, leading to reduced fluorescence signal [29, 33, 35, 44]. Additionally, changes in mitochondrial metabolism decreases fluorescence from flavin adenine nucleotide (FAD) [43]. Increased microvascularization results in higher hemoglobin content [42, 45], increasing absorption of both excitation and emission wavelengths [46]. Lastly, in addition to decreased green wavelength fluorescence, a 635 nm emission peak occurs due to increased porphryin take-up in cancerous cells [47, 48] with the ratio of signal between 635 nm and 500 nm indicating possible cancerous lesions [30, 39, 40].

Previous autofluorescence imaging (AFI) system studies have typically achieved sensitivities of greater than 71% and specificities of 15.3%—100% [30, 42, 49–54] though a few studies have achieved sensitivities of only 30%–50% [45, 55]). Increased sensitivity will lead to earlier diagnosis of oral cancer, enabling prompt treatment of the disease, while the specificity of an AFI device needs to remain high to avoid unneeded, invasive biopsies.

In high-risk, remote populations with low doctor-to-patient ratios, the ideal AFI system is operable by any frontline health worker in primary health centers, dentists, nurses, or by any community member, even those without formal healthcare training. In the cases where a trained specialist is not present, a remote specialist can be integrated into the clinical environment through the internet, allowing for informed diagnosis. Smartphones provide portable image collection, computation, and data transmission capabilities controlled by a simple touchscreen interface, addressing the needs of a cancer screening device being simple to use and connected to the internet. Using the smartphone’s data transmission capabilities, the collected data can be uploaded to a cloud server, where a remote specialist can access the images and make a diagnosis. Additionally, deep-learning tools like a CNN can be implemented in the cloud and used for automatic image analysis and classification [56].

## 2 Materials

### 2.1 Hardware

**Fig 1:**
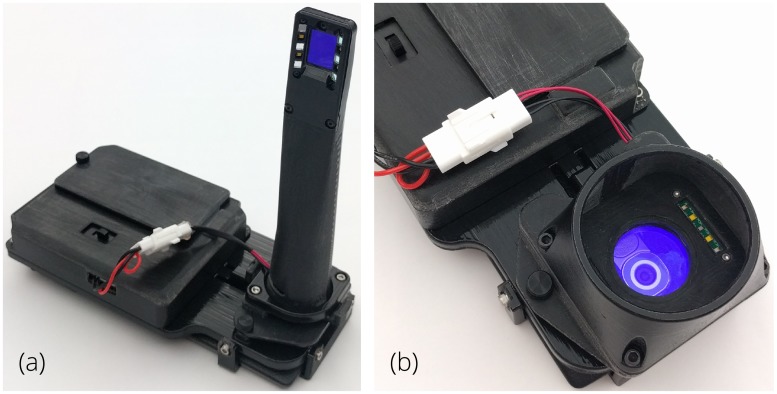
Smartphone-based oral cancer screening device using both WLI and AFI. Interchangeable modules installed on a common platform allow for both (a) intraoral imaging and (b) whole cavity imaging.

To address the need for oral cancer screening in high-risk populations, we have developed a low-cost, point-of-care smartphone-based system (Fig 1). The dual-view, oral cancer screening device augments a commercially available Android smartphone (LG G4, LG, Seoul, South Korea) for AFI and white light imaging (WLI) both internal to the oral cavity with an intraoral probe, and external with a whole mouth imaging module [57]. The whole cavity imaging module provides a wide field of view (FOV) image for assessment of the patient’s overall oral health.

**Fig 2:**
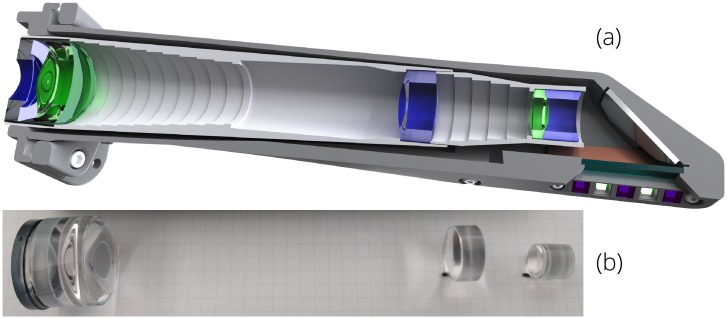
(a) Intraoral probe section view showing the mechanical structure, lenses, and illumination LEDs; and (b) the diamond turned lenses.

**Fig 3:**
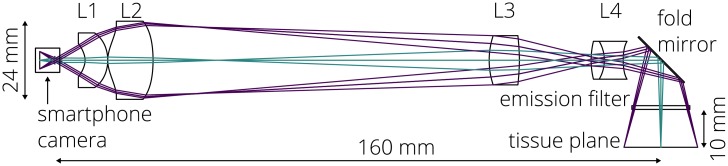
Layout of the intraoral probe optical design.

**Fig 4:**
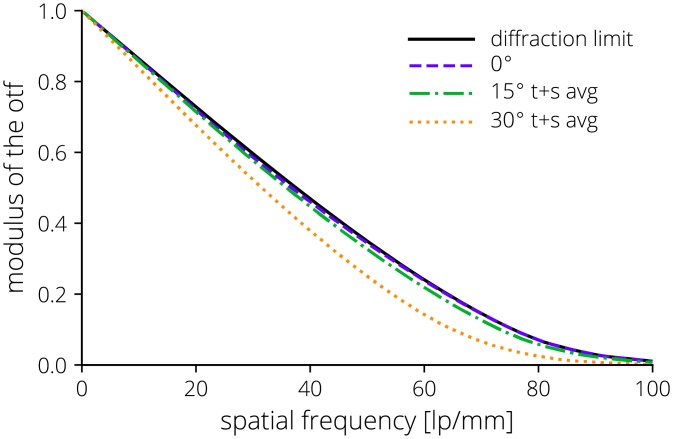
Nominal MTF of the intraoral probe at the tissue plane with the sagittal and tangential data averaged.

The intraoral probe’s custom optical system (Figs 2 and 3) extends the entrance pupil away from the smartphone camera aperture and allows for close-focus imaging of the oral tissues. A hygienic sleeve (TIDI Products, Neenah, WI) is used with the intraoral probe for infection prevention. Smartphone cameras are well-designed to capture a wide field of view from a relatively long distance away, and modifying this optical system to (a) decrease the field of view by ∼90%, (b) focus on a close object, (c) utilize the entire image sensor, and (d) yield a packaged design to fit comfortably in the oral cavity and access base of tongue and cheek pockets is challenging. During the design process, the lenses of the smartphone camera were modeled as a single paraxial surface to ensure compatibility with any smartphone camera whose camera can be set to infinite focus. The prescription of the optical system is provided in Table 1. The sag of the aspheric surfaces is defined using an even polynomial [58]

$$\begin{array}{c} z=\frac{c\;r^{2}}{1+\sqrt{1-\left(1+k\right)\;c^{2}\;r^{2}}}+\alpha_{2}\;r^{4}+\alpha_{3}\;r^{6} \end{array}$$ (1)

where *r* is the radial distance from the optical axis, *c* is the curvature (1/*R*), *k* is the conic constant, and the *α*’s define the coefficients of the even *r*-polynomial. The lenses were designed using poly(methyl methacrylate) (PMMA) and OKP4HT (Osaka Gas Chemicals, Osaka, Japan) and fabricated using single point diamond turning (Moore Nanotechnology Systems, Swanzey, NH). A rendered sectioned view of the intraoral probe assembly and the manufactured lenses are shown in Fig 2. A layout of the optical system is shown in Fig 3 and the nominal modulation transfer function (MTF) is provided in Fig 4.

**Table 1:** Intraoral probe optical system prescription.

Surface	Material	Radius	Thickness	Conic	*α*_2_	*α*_3_
Obj	air	infinity	32.6			
1	OKP4HT	-20.585	5.0	12.222	-3.086·10^-4^	8.902·10^-7^
2	PMMA	9.862	3.5			
STOP	air	-20.904	19.0	15.340	-5.511·10^-5^	5.041·10^-6^
4	PMMA	46.623	8.0	11.035	-4.636·10^-5^	-8.567·10^-8^
5	air	-20.564	89.0	-1.795	-2.443·10^-5^	-5.850·10^-8^
6	PMMA	33.722	12.0	4.506	5.408·10^-5^	1.450·10^-7^
7	air	-7.986	0.0	-4.612	7.972·10^-5^	4.231·10^-7^
8	OKP4HT	10.437	6.0	0.397	3.454·10^-5^	1.445·10^-6^
9	air	4.480	8.0	-3.363	4.041·10^-4^	1.126·10^-5^
10	smartphone camera					

The system utilizes six 405 nm Luxeon UV U1 LEDs (Lumileds, Amsterdam, Netherlands) to enable AFI and four 4000 K Luxeon Z ES LEDs (Lumileds) for WLI and general screening. The LEDs are placed in a plane-symmetrical pattern on either side of the optical axis (Figs 1 and 2). In the intraoral probe, the LEDs are angled toward the object plane to increase illumination uniformity. An emission filter (Asahi Spectra, Tokyo, Japan) with a 470 nm cut-on wavelength is installed in the imaging channel for AFI and excitation filter (Asahi Spectra) is installed in front of the violet LEDs to limit output in the passband of the emission filter. The whole mouth module uses the unmodified smartphone camera optics to provide wide FOV imaging and includes both wavelengths of illumination LEDs, with an emission filter for AFI in the imaging channel.

**Fig 5:**
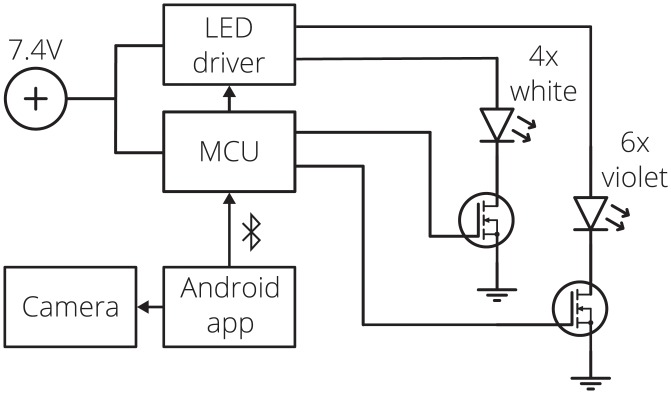
Block diagram of the oral screening system electronics.

The illumination LEDs are driven with a switching boost voltage regulator (Linear Technology, Milpitas, CA) controlled by a custom Android application (Sec 2.2) through a Bluetooth connected microcontroller unit (MCU, SparkFun Electronics, Niwot, CO). Two 3.7 V 18650 Li-ion batteries (Orbtronic, Saint Petersburg, FL) power the MCU and LED driver. The MCU sets the LED current through a digital potentiometer (Analog Devices, Norwood, MA) and switches between the LED strings using signal voltages applied to MOSFETs. The smartphone application synchronizes the LED illumination with image capture, optimizing the LED on-time, reducing power consumption and generated heat. A block diagram is shown in Fig 5.

Finally, the phone and electronics are mounted to a low-cost, 3D-printed mechanical structure of VeroBlackPlus RGD875 plastic (Stratasys, Eden Prairie, MN). This structure also provides a universal mount for the interchangeable imaging modules. A simple redesign of the mechanical structure could allow for a variety of smartphone sizes and camera locations on the backside of the smartphone.

### 2.2 Software

**Fig 6:**
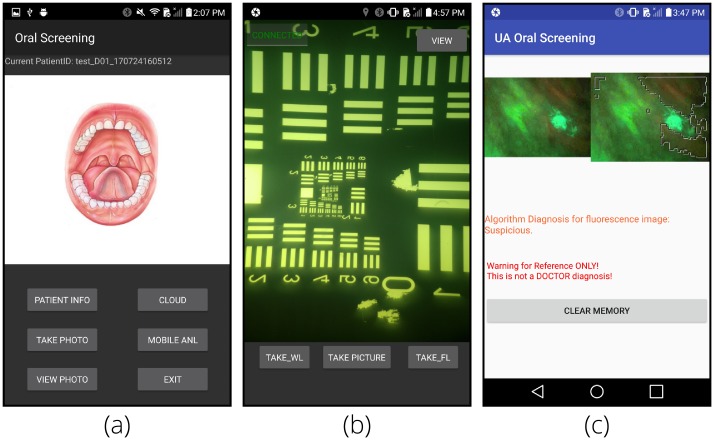
Screenshots of the custom Android application. (a) shows the main menu of the app where buttons allow for navigation to image capture, image viewing, image upload to the cloud, and mobile analysis. (b) shows the image capture interface for both WLI (using the *‘TAKE_WL’* button) and AFI (using the *‘TAKE_FL’* button) individually or sequentially using the *‘TAKE PICTURE’* button. (c) shows a sample result for on-phone image processing of AFI images.

A custom Android application (app) was developed to guide the user through the data collection process. When first opened, the app prompts the user to create a new case ID or select an ID from a previous session, storing all the data from a single patient under the same ID. From the main menu, relevant patient data (age, history of tobacco or paan use, etc.) can be input, AFI and WLI images can be collected and viewed, on-phone image processing can be completed (Fig 6), or data can be uploaded to the cloud. During image capture, the smartphone uses its Bluetooth connection to communicate with the MCU to synchronize image capture and the LED illumination. After image capture, the images may be viewed within the app or the AFI images processed on the phone using the red-to-green signal ratio [30, 59] with a ‘suspicious’ or ‘not suspicious’ classification.

The Android Camera2 API [60] is used to enable low-level camera control by the app, including exposure, gain, focus, ISO, color conversion, and white balance. The LG G4 device runs Android 6.0 Marshmallow which supports most of the Camera2 API features and the Camera2 API is compatible with Android 5.0 Lollipop and newer allowing 84.7% of Android devices to run the app [61]. Additionally, the app could be ported to other popular smartphone operating systems though the device cost could significantly increase.

**Fig 7:**
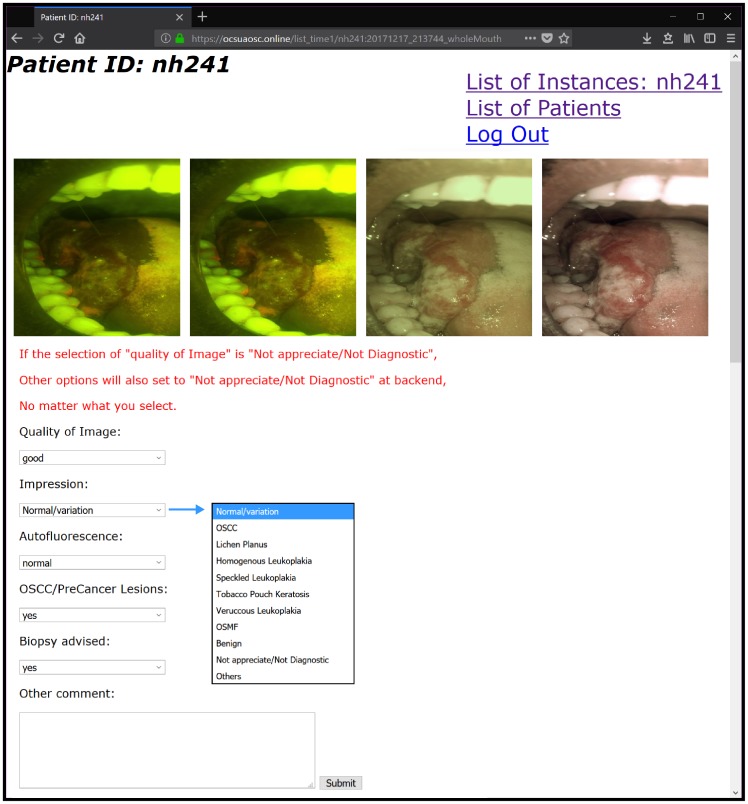
Sample web portal screen for remote viewing and diagnosis of images. The four images presented from left to right are: (i) original AFI, (ii) AFI with histogram equalization, (iii) original WLI, (iv) WLI with color correction.

The patient data, images, and location data (for further spatio-temporal analysis) is uploaded to a cloud server through Wi-Fi and can be remotely accessed anywhere with an internet connection through a web app deployed on the server (Fig 7). When viewing images, the specialist is presented with original, full-resolution images along with sliders to adjust contrast and brightness. On the same web-page the specialist uses dropdown menus to select a diagnosis from list (normal, lichen planus, leukoplakia, erythroplakia, etc.) and a text box to provide triage instructions to the patient.

The cloud server hosts a virtual machine configured on a Google cloud compute engine to automatically classify uploaded images with a pre-trained convolutional neural network (CNN), determining the likelihood of the presence of suspicious lesions in each image.

A reminder email is automatically sent to the remote specialists whenever a new case is uploaded to the cloud. Once a remote specialist diagnoses a waiting case, a summary report is generated with uploaded data from the smartphone, CNN results, and diagnoses. The reports can be viewed continuously on the web app and also be downloaded to the smartphone through the Android app.

## 3 Methods

### 3.1 System characterization

#### 3.1.1 Imaging

Performance of the intraoral imaging system was characterized by (a) measuring the MTF without the smartphone camera, (b) measuring the MTF with the smartphone camera, (c) evaluating the predicted assembled performance with a Monte Carlo analysis, (d) measuring the cutoff frequency, and (e) evaluating the field of view.

The cutoff frequency and field of view of the intraoral probe optical system was validated by imaging a 1951 USAF resolution test chart.

The MTF of the optical system was directly measured using both an Optikos LensCheck (Optikos, Wakefield, MA) instrument and the slanted edge method [62, 63]. The LensCheck system directly measures the point-spread function (PSF) of the intraoral lens system without the smartphone camera lens or image sensor and the MTF is calculated from the normalized Fourier transform of the PSF. The slanted edge method was used to measure the entire optical system including the external lens system, the smartphone camera, and the image sensor. The slanted edge method measures an edge-spread function (ESF) of which the derivative is the line-spread function (LSF). The normalized Fourier transform of the LSF is the one-dimensional MTF. The results from multiple regions of interest across the slanted edge in the central field of view were averaged. The spatial frequency limit was then scaled by the limiting spatial frequency of the added intraoral optical system. Both MTF measurements were compared to representative assembled performance of the passively aligned intraoral probe optics modeled using a Monte Carlo analysis in Zemax OpticStudio (Zemax, Kirkland, WA).

Due to the imaging channel emission filter, the color space is distorted. More accurate color representation is important for image evaluation by a remote specialist and is achieved by applying a custom color matrix

$$\begin{array}{c} \left|\begin{array}{c} X\\ Y\\ Z \end{array}|=|\begin{array}{ccc} a_{11} & a_{12} & a_{13}\\ a_{21} & a_{22} & a_{23}\\ a_{31} & a_{32} & a_{33} \end{array}||\begin{array}{c} R\\ G\\ B \end{array}\right| \end{array}$$ (2)

defined by *a*_*mn*_ values that maps the camera RGB values to the CIEXYZ color space [64]. After imaging a standard 24-patch color checker board (X-Rite, Grand Rapids, MI) with known CIEXYZ values, the **A** matrix composed of the *a*_*mn*_ coefficients can be calculated by

$$\begin{array}{c} A=C^{-1}T \end{array}$$ (3)

where **T** is the matrix of known CIEXYZ values and **C** is the matrix of measured RGB camera values.

#### 3.1.2 Illumination

The white light and violet light illumination uniformity was measured by imaging a matte white surface without the emission filter in place. For this test, the violet LEDs were replaced with white LEDs with similar radiance characteristics from the same product series (Luxeon Z) to avoid exciting fluorescence from the measurement surface. The uniformity measurements are corrected by the relative illumination (RI) of the imaging system. The RI of the combined intraoral lens system and the smartphone camera was measured using a liquid light guide coupled source diffused by multiple plates of ground glass. The measured uniformity is compared to a non-sequential raytracing model (FRED, Photon Engineering, Tucson, AZ) using LED rayfiles from the manufacturer. Uniformity is quantified using the coefficient of variation (*c*_*v*_) [65] on normalized data,

$$\begin{array}{c} \text{Uniformity}=1-c_{v}=1-\frac{\sqrt{\frac{\sum_{i=1}^{N}{\left(x_{i}-\;\bar{\;x\;}\;\right)}^{2}}{N-1}}}{\frac{1}{N}\sum_{i=1}^{N}x_{i}}=1-\frac{\sigma }{\;\bar{\;x\;}\;}, \end{array}$$ (4)

where *x*_*i*_ is the luminance value of each pixel, $\;\bar{\;x\;}\;$ is the mean of the pixels in the image, and *σ* is the standard deviation of the pixel values.

### 3.2 Field testing and CNN classification

A pilot human subjects study was performed at KLE Society’s Institute of Dental Sciences (Bangalore, India), Mazumdar Shaw Medical Centre (Bangalore, India), and the Christian Institute of Health Sciences & Research (Dimapur, India) to demonstrate the feasibility of the oral cancer screening hardware, remote clinical diagnosis workflow, and classification algorithms. This study received institutional review board (IRB) approval from Mazumdar Shaw Cancer Centre (NNH/MEC-CL-2016-394) and University of California, Irvine (HS#2002-2805). All subjects provided informed written and oral consent.

Inclusion criteria included clinically suspicious oral lesions, a history of previously treated OSCC with no current evidence of cancer recurrence at least six months after cessation of treatment, or the presence of recently diagnosed, untreated OSCC or pre-cancerous lesions. Exclusion criteria included being less than or equal to 18 years of age, currently undergoing treatment for malignancy, pregnancy, under treatment for tuberculosis, or suffering from any acute illness.

**Fig 8:**
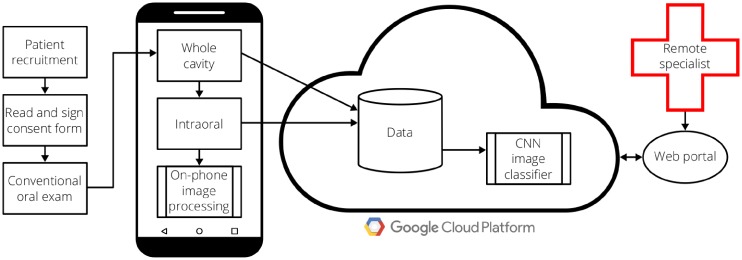
Field testing workflow for smartphone-based oral screening.

The full field testing workflow is shown in Fig 8. When patients arrived for their visit, they first read, understood, and signed a consent form. After acknowledging consent, a general dentist or oral oncology specialist performed a conventional visual oral exam. Following, the general dentist performed the smartphone-based imaging exam, collecting both AFI and WLI with both the whole cavity imaging module and the intraoral probe module. Finally, the oral oncology specialist clinically diagnosed each lesion site, with the clinical diagnosis serving as the gold standard.

Based on the gold-standard diagnosis, the images were assigned to either the normal class or suspicious class. Diagnoses of oral squamous cell carcinoma, lichen planus, homogeneous leukoplakia, speckled leukoplakia, tobacco pouch keratosis, verruccous leukoplakia, and oral submucous fibrosis were included in the suspicious class. Diagnoses of normal/variation were included in the normal class. Variation includes normal variations of oral mucosa, including fissured tongue, Fordyce granules, leukoedema, physiological pigmentation, and linea alba buccalis [66–68]. Diagnoses of benign were not included in either class.

The captured images were uploaded to the cloud server for diagnosis by a remote specialist, and for the intraoral images, classification by the conventional neural network (CNN). Image pairs (WLI and AFI) were screened by the remote specialist for sufficient image quality (minimal motion blur, in focus) to make a diagnosis.

The intraoral images were then classified with a trained CNN. For the CNN training, methods commonly used in network training were applied including transfer learning [69] and data augmentation [70–72]. For data augmentation, the original images were rotated and flipped to feed the network more data for training. Additionally, transfer learning was applied by using a VGG-M [70] network pre-trained on the ImageNet dataset [73]. The network was modified for our task by replacing the final dense layer and softmax layer and then training the network with our dataset.

Sensitivity, specificity, positive predictive value (PPV), and negative predictive value (NPV) [74, 75] were calculated to compare the remote specialist diagnosis and the CNN result to the gold-standard on-site specialist diagnosis. Lastly, a receiver operating characteristic (ROC) curve was generated to determine the accuracy of the classifier and area under the ROC curve (AUC) calculated to provide a single value for comparison to other devices [76, 77].

## 4 Results

### 4.1 System performance

#### 4.1.1 Imaging

**Fig 9:**
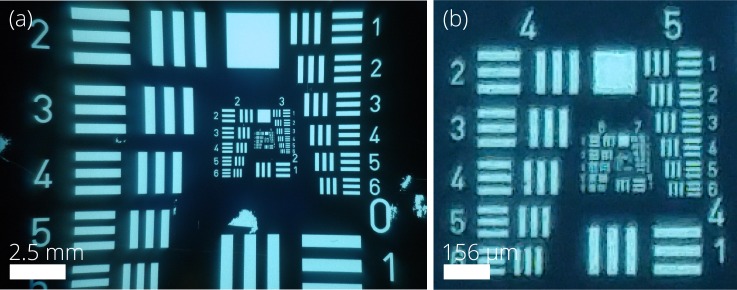
Image of a 1951 USAF resolution test chart showing the (a) full field of view of the intraoral probe and (b) contrast limit. The zoomed contrast limit image (b) shows group 6–2 is resolvable, a cutoff frequency of 71.8 lp/mm.

Fig 9 provides the resulting image of a 1951 USAF resolution test chart, showing a resolution limit of 71.8 lp/mm and also the full field of the view of the intraoral probe.

**Fig 10:**
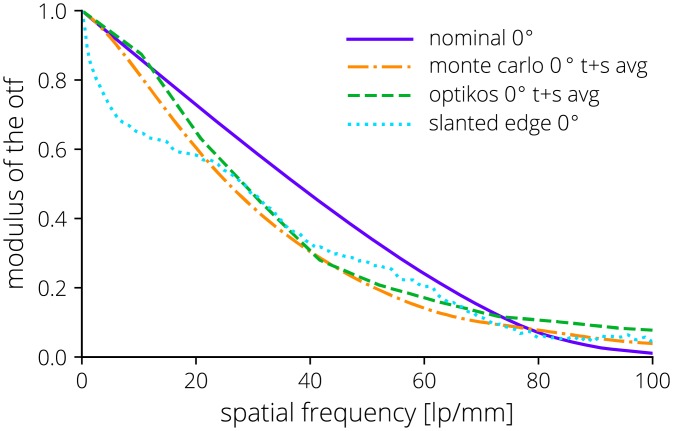
Comparison of the nominal, Monte Carlo, and measured on-axis MTF performance. The Monte Carlo analysis is an average system output. Measured MTF data is from an Optikos LensCheck instrument and from a slanted edge test. The sagittal and tangential data has been averaged where noted.

The measured MTF along with the performance of an average system from the Monte Carlo analysis with representative tolerances is shown in Fig 10. A sensitivity analysis shows decenter of the outside concave surface of L4, L3 decenter, and L4 decenter have the greatest effect on performance.

Lastly, the color mapping **A** matrix was calculated to be

$$\begin{array}{c} A=|\begin{array}{ccc} 0.81795 & 0.09584 & -0.02293\\ -0.11767 & 0.98376 & 0.07395\\ -0.19637 & 0.57655 & 2.28612 \end{array}| \end{array}$$ (5)

#### 4.1.2 Illumination uniformity

Data for the modeled and measured uniformity for the intraoral and whole mouth modules are provided in Table 2.

**Table 2:** Measured and modeled uniformity for white light and violet light for the intraoral probe and whole mouth module. The measured uniformity is adjusted by the relative illumination of each optical system.

Color	Intraoral		Whole cavity	
	Modeled	Measured	Modeled	Measured
White	0.85	0.92	0.94	0.96
Violet	0.89	0.93	0.95	0.96

### 4.2 Field testing and CNN classification

Data was collected at the three testing sites from 190 patients with data from 99 patients (demographics shown in Table 3) used for CNN analysis and remote diagnosis.

**Table 3:** Study participant demographics for the image pairs used in CNN classification. Values are provided as the *N* of each category except for age in units of years. The *Both* health behavior represents the combination of both smoking and chewing. Health behavior was not collected for all participants.

Item	Female	Male	Total
*N*	46	53	99
Age, *μ* [yr]	37.4	42.2	40.0
Age, *σ* [yr]	15.0	13.0	14.1
*Health behavior*			
None	8	6	14
Smoking	0	7	7
Chewing	17	25	42
Both	3	14	17
Alcohol	3	9	12
*Clinical diagnosis*			
Normal	24	9	33
Lichen Planus	2	6	8
Homogeneous Leukoplakia	6	10	16
Speckled Leukoplakia	1	2	3
Tobacco Pouch Keratosis	12	21	33
Squamous Cell Carcinoma	1	5	6

**Fig 11:**
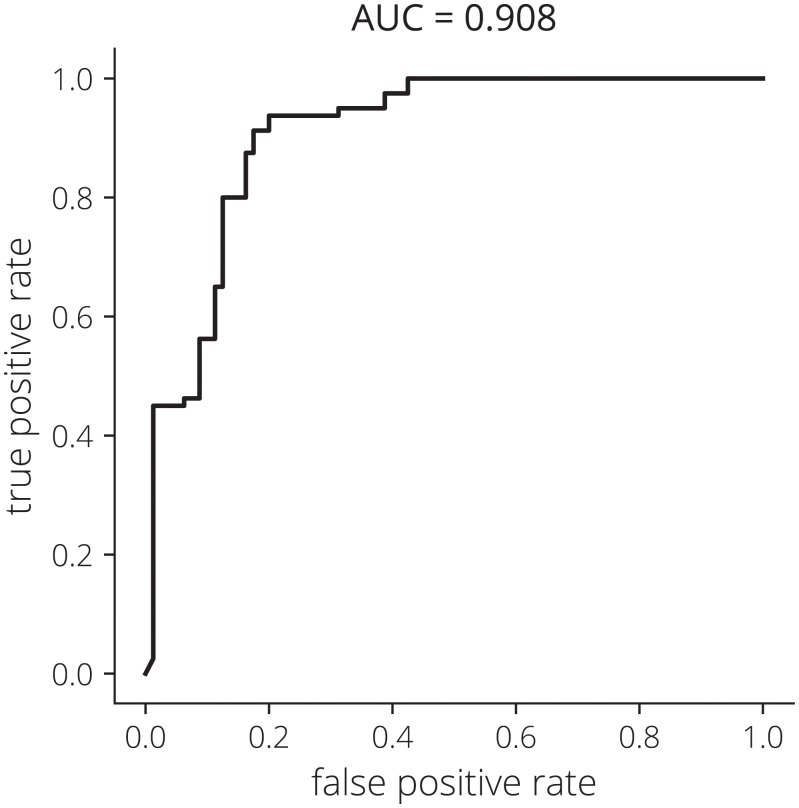
Receiver operating characteristic (ROC) curve for the CNN. The area under the curve (AUC) equals 0.908.

Out of 364 image pairs, 170 WLI and AFI image pairs had sufficient quality for remote diagnosis and use in the CNN with *N* = 86 in the normal class and *N* = 84 in the suspicious class. Data augmentation increased the dataset size by 8× to 1360 image pairs. After training the network for 80 epochs, four-fold cross validation accuracy of the VGG-M network was 86.88%. The ROC curve for the CNN is provided in Fig 11 and the AUC = 0.908.

Sensitivity, specificity, PPV, and NPV values comparing the remote specialist diagnosis and the CNN result to the gold-standard on-site oral oncology specialist clinical diagnosis are provided in Table 4.

**Table 4:** Sensitivity, specificity, PPV, and NPV values for the images of sufficient quality for remote diagnosis and CNN evaluation compared to the gold-standard on-site specialist clinical diagnosis.

Parameter	Remote specialist	CNN
Sensitivity	0.9259	0.8500
Specificity	0.8667	0.8875
PPV	0.9494	0.8767
NPV	0.8125	0.8549

**Fig 12:**
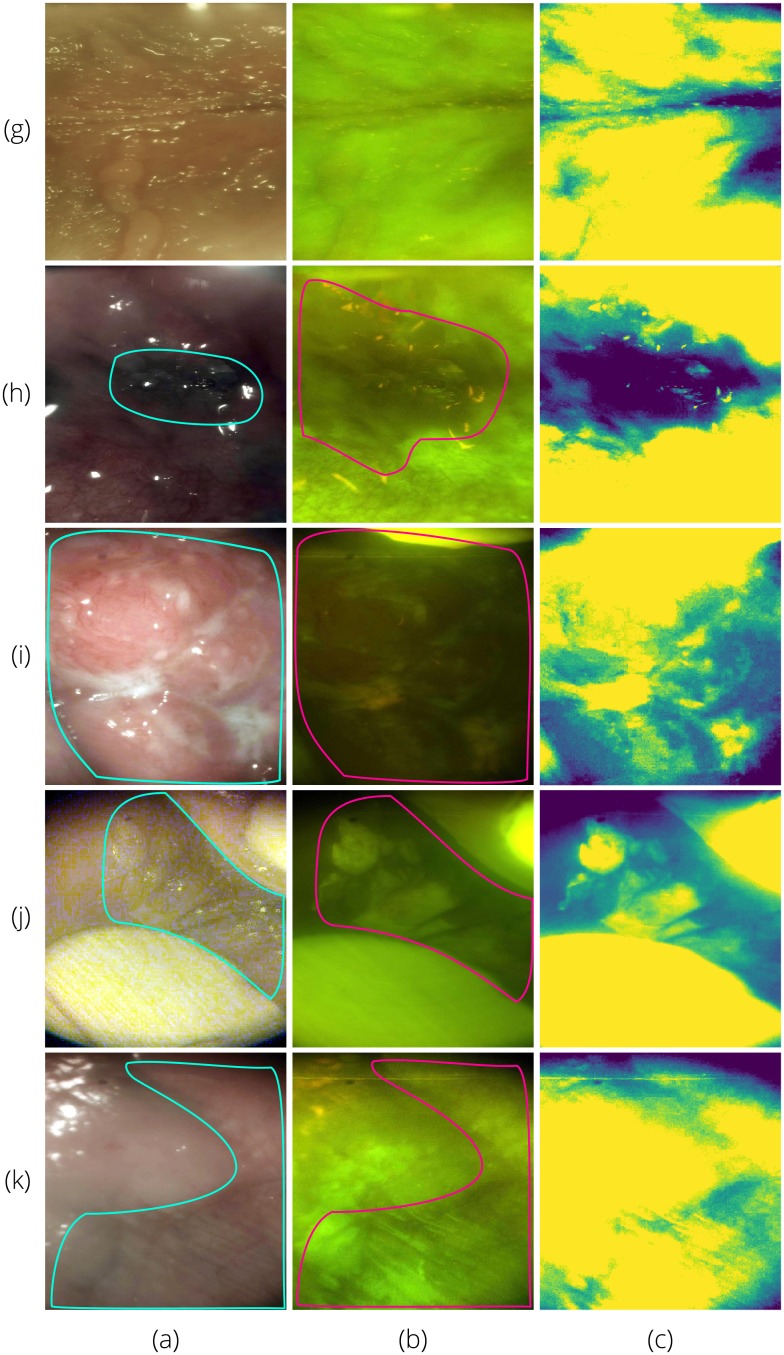
Sample white light (column a) and autofluorescence (column b) intraoral probe field testing images with suspect areas outlined. All rows were classified by the CNN as *suspicious*. On-site specialist diagnoses were: (g)—normal/variation; (h)—homogeneous leukoplakia; (i)—carcinoma of the left mandibular alveolus; (j)—tobacco pouch keratosis demonstrating increased fluorescence due to hyperkeratosis; and (k)—tobacco pouch keratosis. Column (c) shows the green intensity map with the mean subtracted as discussed in Section 5.

**Fig 13:**
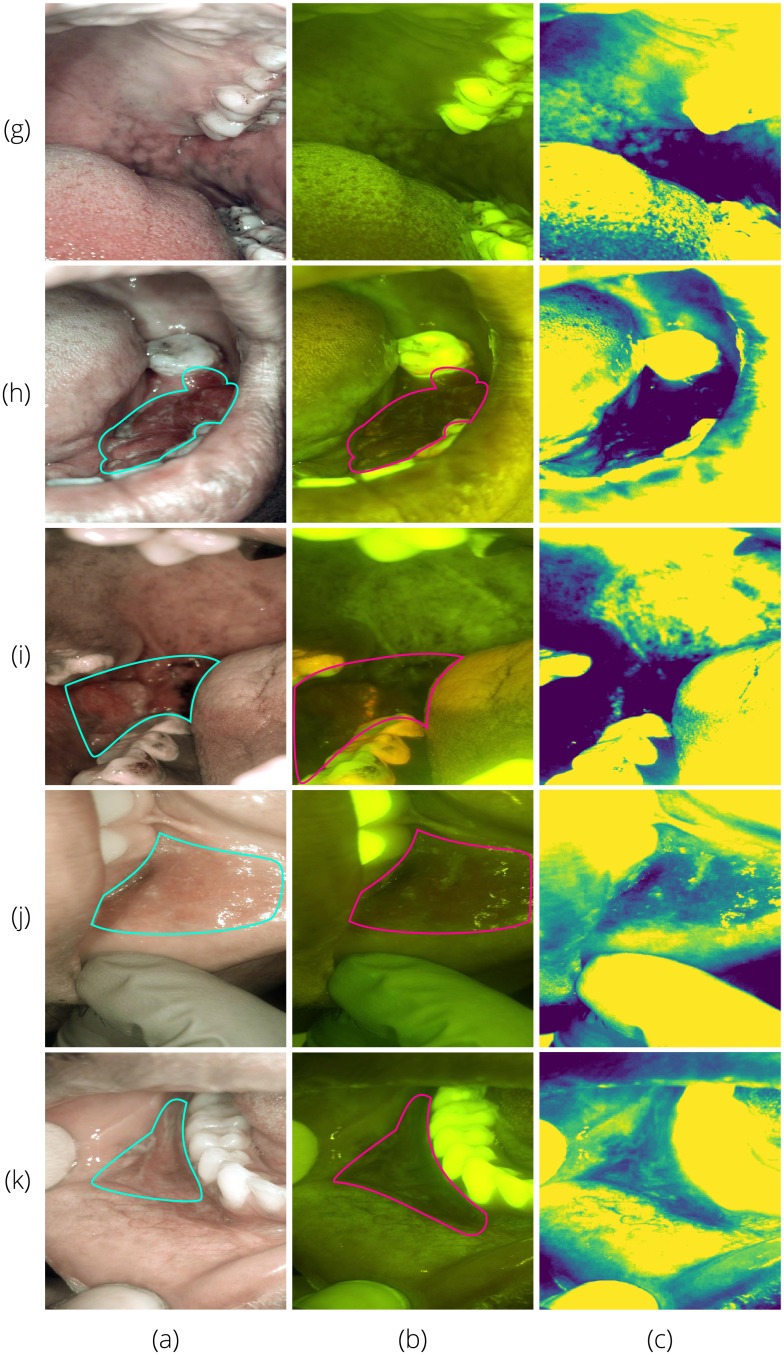
Sample white light (column a) and autofluorescence (column b) whole cavity module field testing images with suspect areas outlined. All rows were classified by the CNN as *suspicious*. On-site specialist diagnoses were: (g)—normal/variation; (h)—carcinoma of the left mandibular alveolus; (i)—oral squamous cell carcinoma; (j)—tobacco pouch keratosis; and (k)—homogeneous leukoplakia. Column (c) shows the green intensity map with the mean subtracted as discussed in Section 5.

Sample images diagnosed by a remote specialist are shown in Figs 12 and 13. Fig 12 shows AFI and WLI taken with the intraoral probe. With suspect areas outlined, the combination of WLI and AFI provides the most information about the type of lesion and the size of the affected area. Fig 13 provides similar findings for the whole cavity imaging module.

## 5 Discussion

The smartphone platform is a natural progression of previous autofluorescence systems targeting oral lesions [34, 41, 42, 78–83] and our device offers several improvements. Compared to previous smartphone-based systems [83], the two FOVs are useful for both an overview of the oral cavity health along with targeted imaging of problem areas. Our intraoral probe extends capability, reaching to the base of the tongue and cheek pockets in some patients, areas of increased cancer risk [4]. Our device offers image capture, save, review, and transmit of both AFI and WLI captured both intraorally and externally to the oral cavity. Additionally, our intraoral imaging attachment utilizes a custom designed optical system to maximize the number of pixels used on the smartphone image sensor. Operation of the system is simple through an intuitive user interface. Since the device is connected to the cloud and remote diagnosis is possible, the system does not need to be operated by a specialist, with the remote specialist integrated into the clinical environment through the internet. Importantly, the device implements a machine learning algorithm to aid both the community health workers and the remote specialists as devices requiring the human visual system (HVS) to make decisions based on small changes in scene or image brightness are suboptimal due to the logarithmic response of the HVS [84, 85].

The measured imaging performance of the device matches the predicted performance for a passively aligned optical system and is sufficient for an oral cancer screening device, able to resolve features down to 14 μm. Similar to the Monte Carlo result, the measured mid-spatial frequency performance is decreased from the nominal. Contributions to the decreased performance include stray light from various mechanical surfaces, chromatic aberration, and passively aligned lenses. The TIDI Products SureClear Window is specifically designed to minimally affect image quality through the sheath, though the barrier can increase aberrations and specular reflection from the white LEDs when saliva is introduced on the barrier. Image sensor noise and the proprietary image processing pipeline of the smartphone have the opportunity to decrease the resolution cutoff of the optical system. Automatic, immutable image processing implemented by the smartphone manufacturer including edge sharpening could explain differences between the Optikos and slanted edge measured results. Additionally, single-point diamond turning tool marks cause diffraction-type scatter proportional to the power spectral density (PSD) of the surface, diminishing the quality of the PSF [86]. Due to the low amount of nominal distortion (<0.8%) in the optical system, distortion is not calibrated to save computation time and power in the system.

The measured and modeled illumination uniformity match well for both modules and illumination wavelengths. The whole cavity module uniformity error is only 2% for white illumination and 1% for violet illumination. For the intraoral probe the error is slightly larger at 7% for white light illumination and 4% for violet illumination. The increase in uniformity from the model is likely due to errors in the scattering properties of the various surfaces in the model, including the system mechanics and the target surface.

Our initial field-testing workflow and results were positive. Through the web app, doctors were able to diagnose cases quickly and efficiently, with the AFI and WLI from two FOVs providing the needed information. Compared to the on-site specialist, the remote specialist was able to correctly diagnose patients as having suspicious lesions with high specificity, sensitivity, and PPV, though the remote specialist’s ability to correctly clear patients without suspicious lesions could be improved. The sensitivity and specificity of previous autofluorescence-only devices can have large variation [54], while also needing to be operated by a specialist. The combination of AFI and WLI in our device should set the sensitivity floor at 60%, the value for a conventional visual exam [27].

The CNN sensitivity, specificity, PPV, NPV, and AUC results are promising given the small size of the dataset, however, future research will need to include benign cases in the training and classification processes. Our AUC value is similar to the high-end of results obtained with similar systems in discriminating healthy tissue from lesions [30, 38, 45, 50, 87], however, results have been mixed and the addition of benign lesions decreased the AUC significantly [38].

Additionally, a study including biopsy and a histopathology gold standard is needed to fully correlate the CNN result. Importantly for our small dataset, data augmentation increased the number of images pairs by 8×, and since the images have no natural orientation, flipped and rotated images are still valid. As improvements to the device are made and the health providers acquire additional time and training with the device, the dataset size and percentage of quality images will increase, leading to improvements in CNN training. We hope augmenting the WLI with AFI and the CNN classification algorithm leads to true diagnostic performance in line with our reported CNN result.

The main challenges to using AFI and WLI for cancerous and pre-cancerous lesion detection include increased fluorescence signal from hyperkeratinization of pre-malignant lesions causing an increase in autofluorescence signal [88] and differentiating between pre-cancerous lesions and areas of inflammation or irritation that can confound either a human or computer diagnosis [42], though combining WLI and AFI with longitudinal data discriminates dysplasia from short-term inflammation. The main challenges to large-scale implementation of this device will be addressing the needs of regions without cellular data or internet access and the additional time burden on the remote specialists for diagnosing cases and monitoring lesion progress. However, the overall time burden should decrease as other community members will be able to collect the necessary data.

Improvements for the next generation device could include the addition of a simple mean subtraction from the green channel of the original AFI

$$\begin{array}{c} f\left(i,j\right)=I_{G}\left(i,j\right)-\frac{\sum_{i=0}^{N-1}\sum_{j=0}^{M-1}I_{G}\left(i,j\right)}{NM}=I_{G}\left(i,j\right)-\;\bar{\;I_{G}\;}\;\left(i,j\right) \end{array}$$ (6)

to the AFI image already presented to provide the diagnosing specialist with an additional map of areas of decreased fluorescence signal as shown in Figs 12 and 13. The on-phone red/green ratio image analysis could also be added to the information shown to the remote specialist (and on-site specialist if present during data collection) [30]. Additionally, including the whole cavity images in the CNN training and classification would increase the amount of data available, however, these images have many additional noise features such as the perioral epidermis and teeth.

A smaller profile for the intraoral probe would be more effective in accessing sites deep in the oral cavity like the cheek pockets and base of tongue, particularly in patients with advanced oral submucous fibrosis. The remote specialist could be better integrated into the clinical environment with a wider field of view and longer depth of field of the intraoral probe to improve area recognition and image quality, helping to orient the remote specialist during diagnosis. Crossed polarizers for the white light LEDs would reduce noise in the image due to specular reflection. Lastly, as use hours increase, app feedback will be used to further streamline the user experience, making data collection easier for all types of users.

Though the targeted communities lack healthcare infrastructure, many have ample cellular data coverage, and as the cost of smartphones continues to decrease, ownership in LMIC increases (the compound annual growth rate (CAGR) of mobile subscriptions in LMICs since 2008 is 20% [89] and the CAGR of smartphone ownership from 2013–2015 is >30% [90]). Smartphone-based devices allow for a hub and spoke model where the hub houses the specialists and trained healthcare workers implementing the screening program and the smartphones extend spokes out to the remote communities. A low system cost enables this model and high-volume cost estimates for our system are ~ $100 plus the cost of the smartphone (The cost of the smartphone is not included since most users will be able to use their own smartphone), an inexpensive medical imaging device.

## 6 Conclusion

Described is the design and implementation of a low-cost, point-of-care, smartphone-based, dual-modality imaging system for oral cancer screening in LMIC. The device enables clinicians and community members to capture AFI and WLI and upload images to the cloud for both remote specialist diagnosis and CNN classification. We have tested the device and diagnosis workflow in three locations in India and initial feedback on the system is positive, with both the remote specialist and CNN achieving high values of sensitivity, specificity, PPV, and NPV compared to the on-site specialist gold standard.

Inexpensive, high-power LED sources in white and violet wavelengths, plastic lens molding technology, and low-cost but powerful smartphones are promising developments for the creation of low-cost, portable, simple-to-use autofluorescence imaging devices for oral cancer detection. Performance should increase as additional images are collected and with improvements to the device hardware and usability. Enabling oral cancer detection in low-resource communities will lead to earlier detection and diagnosis, minimizing disease progression and ultimately, a reduction in oral cancer death rates and healthcare costs.

## 7 Supplemental material

The design files for the LED driver have been released on GitHub under the GPL-3.0 license [91, 92] and the corresponding data repository is found at [93].
